# Alternative splicing of mouse transcription factors affects their DNA-binding domain architecture and is tissue specific

**DOI:** 10.1186/gb-2004-5-10-r75

**Published:** 2004-09-30

**Authors:** Bahar Taneri, Ben Snyder, Alexey Novoradovsky, Terry Gaasterland

**Affiliations:** 1 The Laboratory of Computational Genomics, The Rockefeller University, New York, NY 10021, USA; 2 Scripps Institution of Oceanography, University of California San Diego, La Jolla, CA 92093, USA

## Abstract

**Background:**

Analyzing proteins in the context of all available genome and transcript sequence data has the potential to reveal functional properties not accessible through protein sequence analysis alone. To analyze the impact of alternative splicing on transcription factor (TF) protein structure, we constructed a comprehensive database of splice variants in the mouse transcriptome, called MouSDB3 containing 461 TF loci.

**Results:**

Our analysis revealed that 62% of these loci in MouSDB3 have variant exons, compared to 29% of all loci. These variant TF loci contain a total of 324 alternative exons, of which 23% are in-frame. When excluded, 80% of in-frame alternative exons alter the domain architecture of the protein as computed by SMART (simple modular architecture research tool). Sixty-eight % of these exons directly affect the coding regions of domains important for TF function. Seventy-five % of the domains affected are DNA-binding domains. Tissue distribution analyses of variant mouse TFs reveal that they have more alternatively spliced forms in 14 of the 18 tissues analyzed when compared to all the loci in MouSDB3. Further, TF isoforms are homogenous within a given single tissue and are heterogeneous across different tissues, indicating their tissue specificity.

**Conclusions:**

Our study provides quantitative evidence that alternative splicing preferentially adds or deletes domains important to the DNA-binding function of the TFs. Analyses described here reveal the presence of tissue-specific alternative splicing throughout the mouse transcriptome. Our findings provide significant biological insights into control of transcription and regulation of tissue-specific gene expression by alternative splicing via creation of tissue-specific TF isoforms.

## Abstract 2

Splice variants of 461 transcription factor loci were analyzed using a new database of splice variants in the mouse transcriptome, MouSDB3, providing quantitative evidence that alternative splicing preferentially adds or deletes domains important to the DNA-binding function of the TFs.

## Background

Alternative splicing is a widespread mechanism involved in regulation of gene expression, which enables production of many structurally and functionally different forms of proteins from a single gene, adding to the complexity of the genomes [1-3]. Different mRNA transcripts of a gene can be expressed in different tissues or developmental stages or physiological conditions [4,5].

An expanding body of expressed sequence data from the human and mouse genomes indicates that alternative splicing is an important mechanism in creating protein diversity, and adds to functional complexity encoded in eukaryotic genomes. Earlier studies indicate that at least 50% of the genes in the human genome are alternatively spliced [6]. Examples include the vast majority of immune system and nervous system genes [7].

Comprehensive analysis of alternative splicing is essential to understand fully the proteomes of organisms [8]. Several reports have indicated that variant splice forms result in proteins with different functions. These can range from minimal changes in function to absolutely opposite functions. For example, the cAMP-response element modulator has three different isoforms with entirely different DNA-binding domains, which are all transcription activators. On the other hand, isoforms of the human transcription factor AML1 function both as positive and as negative regulators of transcription [9]. However, for the majority of genes, the functional significance of alternative splicing is still not known [8].

Transcription is a critical process that specifies the mRNAs and the proteins expressed within a cell. Expression of a given gene is dependent on the interactions of different transcription factors and their cofactors with the regulatory regions of that gene. These transcription factors are in turn regulated by processes that include interaction with other proteins and signaling cascades [9].

Alternative splicing is a mechanism that regulates transcription factor (TF) activity by generating a variety of protein isoforms from a single gene. As noted by Lopez, alternative splicing can affect TF structure in two primary ways [9]: alterations can be in the DNA-binding domains affecting their affinity or specificity; or alterations can modulate interactions of transcription factors with their cofactors. Such changes have been observed experimentally to alter specificity or binding strength or to switch between activator and repressor isoforms of the same TF [10]. TF isoforms can have stage-specific and tissue-specific expression patterns throughout the development of an organism [9]. Little is known about the tissue specificity of alternative splicing [11].

In this paper, we use an integrated approach to analyze DNA and protein sequence data jointly to determine the potential effect of alternative splicing on protein structure and function. We perform a detailed analysis of tissue-specific distribution of alternatively spliced mouse TFs to gain biologically meaningful insights into regulation of gene expression by alternative splicing.

## Results

### Definitions

For our joint DNA-protein analysis described here, we developed MouSDB3 [12], which identifies, classifies, computes, stores and answers queries about splice variants within the mouse genome. As described in Materials and methods, MouSDB3 uses the mouse genome and expressed sequences in GenBank [13] and dbEST [14] to compute splice variants of mouse transcripts organized by genomic loci. This section provides definitions of terms used in MouSDB3 and in the joint DNA-protein analysis method described here. A 'transcript' is a sequence transcribed from the genomic DNA sequence. MouSDB3 is restricted to transcripts with at least one splice junction. A 'locus' is a genomic region that includes a set of overlapping transcripts mapped to the genome such that a transcript appears in only one locus and all transcripts whose genome coordinates overlap by at least one nucleotide are included in the locus. Within a locus, a 'cassette exon' is completely included in some transcripts and completely excluded in others. A 'length variant exon' has alternative 5' or 3' splice sites, or both, in different transcripts. An exon can be both length variant and cassette. A 'variant exon' is either cassette or length variant or both. We consider an exon whose number of nucleotides is a multiple of three and which starts at the first base of a codon to be an 'in-frame exon'. Such exons do not introduce an amino-acid substitution or a stop codon when skipped, unless they are terminal exons within the coding sequence. A 'genomic exon' is an uninterrupted series of nucleotides, each of which is mapped to a transcript. By this definition the genomic exon for a length variant exon reflects the outermost splice sites. A 'cluster' is the set of transcripts that map to a locus. A 'variant cluster' contains one or more variant exons. An 'invariant cluster' has no variant exons.

### MouSDB3 cluster analysis

Our cluster analysis revealed that out of the 461 TF clusters, 62% are variant, compared to 29% of all genes in MouSDB3 (Table 1). The majority (62%) of the variation in TFs is due to cassette exons, which is comparable to cassette-exon distribution in the entire transcriptome (68% of the variant exons in all loci are cassette). As the majority of alternative splicing is due to cassette exons, we focus on these exons for our analyses.

### Cassette exon analysis

**Figure 1:**
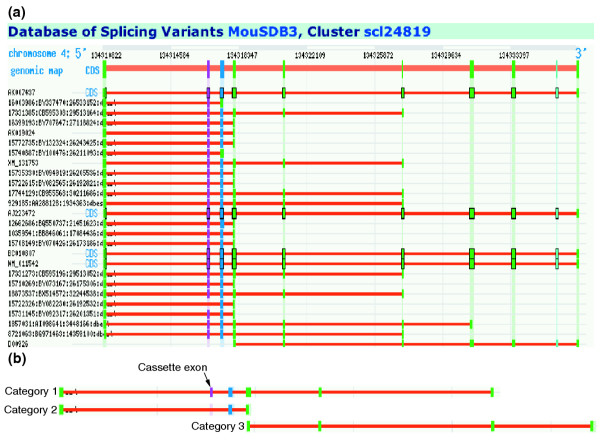
Transcripts of a MouSDB3 cluster. **(a) **Partial image of MouSDB3 cluster number scl24819 [31] displaying alternatively spliced transcripts. **(b) **Categorization of transcripts with respect to the cassette exon indicated by the arrow. This figure shows an example transcript for each of the three categories from the scl24819 cluster. Category 1, long transcript with cassette exon indicated by the arrow. Category 2, short transcript skips the cassette exon. Category 3, cassette exon is missing owing to a 5' truncation. Pink bars represent in-frame cassette exons. Green and blue bars represent exons with other types of splice variation. (Green are invariant, blue are length-variant exons). The red line represents intronic regions of the genome sequence.

We screened the 287 variant TF clusters for the presence of cassette exons within coding sequences. We categorized MouSDB3 transcripts into three categories with respect to each cassette exon within a cluster. Category 1 transcripts contain the exon and are referred to as 'long transcripts'. Category 2 transcripts skip the exon and are referred to as 'short transcripts'. Category 3 transcripts do not overlap with the cassette exon due to 5' or 3' truncations. In our structural analysis, we computationally delete in-frame cassette exons from Category 1 transcripts to produce an 'altered transcript'. Figure 1 displays a MouSDB3 cluster and illustrates these categories.

The 287 variant TF loci contain 324 cassette exons of which 23% (76 exons) are in-frame. Only 11% of cassette exons are expected to be multiples of three and in codon position 1 randomly. The twofold difference between expected and observed numbers indicates a bias towards in-frame cassette exons. The exons which are a multiple of three and in codon position 2 and 3 comprise 10% and 7%, respectively. When deleted, these exons introduce an amino-acid substitution to the sequence. As exons which are a multiple of three starting at codon position 1 are enriched and do not introduce an amino-acid substitution when deleted, our study focuses on these exons only.

**Figure 2:**
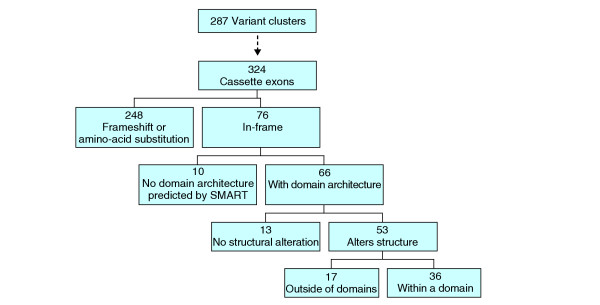
Transcription factor cassette exon analysis. This figure illustrates the distribution of 324 cassette exons within variant TF transcripts. These 324 exons are from 287 different variant MouSDB3 clusters. When 76 of the 324 cassette exons are skipped, the altered transcripts are in-frame; exclusion of remaining exons either introduces an amino-acid substitution or causes frameshifting. Of the in-frame exons, 53 alter domain architecture and 13 do not. Of the exons that cause domain alteration, 36 are in coding regions for domains and 17 are proximal to these coding regions. In-frame cassette exon sequences, sequences of their transcripts and annotations of the domains they alter are provided on our web page [19].

As shown in Figure 2, of the 76 in-frame cassette exons, 66 have domain architectures predicted by SMART. The remaining 10 exons are either from transcripts with too short sequences or these transcripts do not have any of the domains annotated in SMART. Of the 66 in-frame cassette exons, 80% (53) induce a domain-structure alteration to the protein when skipped. Of these 53 structure-altering exons, 68% are within coding regions for the domains that are important for TF activity, such as DNA-binding or activation domains. The remaining 32% (17) of exons are proximal to the computed domain boundaries; that is, the domain is coded by the upstream or the downstream neighboring exon of the cassette exon. When the cassette exon is removed, the sequence no longer meets the computational criteria for the domain (Figure 2).

### Assessing domain architecture alterations

SMART [15,16] and Pfam [17,18] entries for the altered domains revealed that 75% of the domains affected by alternative splicing with known functions are DNA-binding domains. The names of all altered domains and links to their annotated biological functions are provided on our web page [19]. There we provide the 53 in-frame cassette exons (shown in Figure 2), which alter the domain architecture of their transcripts when skipped. Links to MouSDB3 clusters containing these transcripts and links to their GenBank entries are provided. In addition, we provide the names of the domains altered by these 53 exons as active links to their SMART and Pfam annotations. All sequences for long transcripts, altered transcripts and in-frame cassette exons are provided as links to fasta files on the same web page. Our domain-alteration results correlate with recent findings of Resch *et al*. [20], who show that alternative splicing preferentially removes certain domains more frequently.

### Tissue-distribution analysis

Part two of our analysis assessed the tissue distribution of alternatively spliced transcription factors. We chose 18 tissues from the existing libraries in MouSDB3 on the basis of the fact that they contain both variant and invariant transcripts annotated as TFs. There are a total of 1,413 library names in MouSDB3 imported from expressed sequence records in GenBank and dbEST. Of these, 328 are ambiguous in that they list several different tissues or cell types for a single library, such as 'mixture of brain and testis' or no tissues at all, such as 'embryo or carcinoma'. For the work described here we did not include tissue information from such ambiguous libraries. There are a total of 95 libraries in MouSDB3 for which there are TF transcripts. In addition, to account for library ambiguities within these 95 libraries, we pooled different parts of a tissue into one library. For example, the term 'brain' corresponds to all parts of the brain found in MouSDB3, including cerebellum, thalamus, hippocampus and 16 other libraries. When analyzing the tissue distribution of all genes, only the libraries that contain TF transcripts have been used.

**Figure 3:**
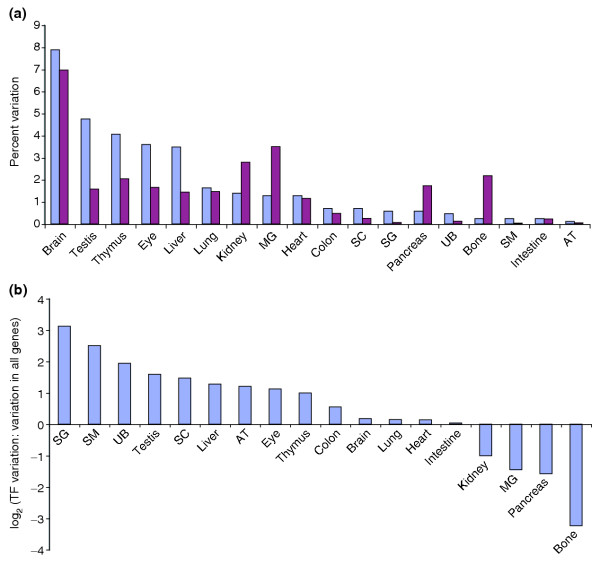
TF variation is higher in the majority of tissues compared to all genes. **(a) **Tissue distribution of alternatively spliced TFs versus tissue distribution of all alternatively spliced genes. For each tissue, the number of variant TF transcripts in tissue normalized by the total number of variant TF transcripts in MouSDB3 is represented as a blue bar. This number is computed as follows: *t *= number of variant TF transcripts in tissue; *T *= total number of variant TF transcripts; bar value = (*t*/*T *× 100). Red bars represent the number of variant transcripts of all genes in the tissue normalized by the total number of variant transcripts in MouSDB3. This value is computed as follows: *a *= total number of variant transcripts in tissue; *A *= total number of all variant transcripts in MouSDB3; bar value = (*a*/*A *× 100). **(b) **Fold differences in variant number of transcripts between TFs and all genes. This value is computed as follows: bar value = log_2_((*t*/*T*)/(*a*/*A*)). Tissues are in descending order from highest to lowest fold difference of variation in TF versus variation in all genes. Tissue abbreviations: SG, salivary gland; SM, skeletal muscle; UB, urinary bladder; SC, spinal cord; AT, adipose tissue; MG, mammary gland.

Transcript counts within variant loci for 18 pooled libraries indicated that in 14 of the 18 analyzed tissues, the proportion of TFs that are variant is higher than the proportion of all genes that are variant (Figure 3a). This finding, together with the observation that 62% of TF loci are variant, indicates the widespread impact of alternative splicing on regulation of gene expression via TFs.

**Figure 4:**
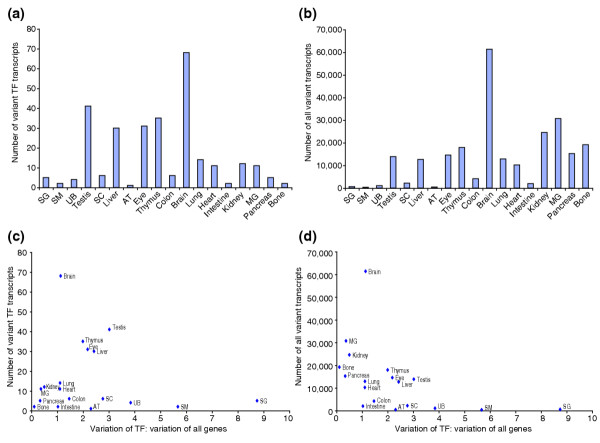
Higher variation in TFs is independent of sampling depth from each tissue. **(a) **Absolute number of variant TF transcripts per tissue. **(b) **Absolute number of all variant transcripts per tissue. **(c) **For each tissue (labeled to the right of each data point), *x*-axis: ratio of variant TF transcripts to all variant transcripts (*x *= (*t*/*T*)/(*a*/*A*)); *y*-axis: absolute numbers of variant TF transcripts. See Figure 3 legend or definitions of *t*, *T*, *a *and *A*. **(d) **For each tissue (labeled to the right of each data point), *x*-axis: ratio of variant TF transcripts to all variant transcripts (*x *= (*t*/*T*)/(*a*/*A*)); *y*-axis: absolute numbers of all variant transcripts. Tissue abbreviations: SG, salivary gland; SM, skeletal muscle; UB, urinary bladder; SC, spinal cord; AT, adipose tissue; MG, mammary gland.

For each of the 18 tissues in Figure 3a, we compared the proportion of TFs that vary to the proportion of all genes that vary. As shown in Figure 3b, eight tissues exhibited more than twofold difference in variant TFs versus variant genes in total. (Note that values in Figure 3b are base 2 logarithms of the ratios. Tissues with twofold differences have log_2 _values above 1 on the graph). In salivary gland, skeletal muscle, urinary bladder and testis, the fold-differences are 8.7, 5.6, 3.8 and 3.0-fold respectively. Spinal cord, liver, adipose tissue and eye also exhibit more than twofold differences. These values are independent of the sampling depth of the transcripts from these tissues, as illustrated in Figures 4a and 4b. Sampling depth is the number of transcripts sequenced per tissue (either a single library or a pooled library as in the case of 'brain'). Figure 4a displays absolute numbers of variant TF transcripts and Figure 4b displays absolute numbers of the entire variant transcripts of the transcriptome. In Figures 4a,b, tissues are presented along the *x*-axis as in Figure 3b for the reader's convenience. The correlation coefficient of the absolute numbers of TFs and the fold-differences between variant TFs and all genes is -0.13, indicating that they do not correlate. Likewise, the correlation coefficient of the absolute numbers of all genes and the fold-differences between variant TFs and all genes is -0.46. Additionally, the scatter-plots in Figures 4c,d show that there is no correlation between the fold-differences and sampling depth. The datasets used in calculating the correlation coefficients can be found on our web page [19].

## Isoform heterogeneity

**Figure 5:**
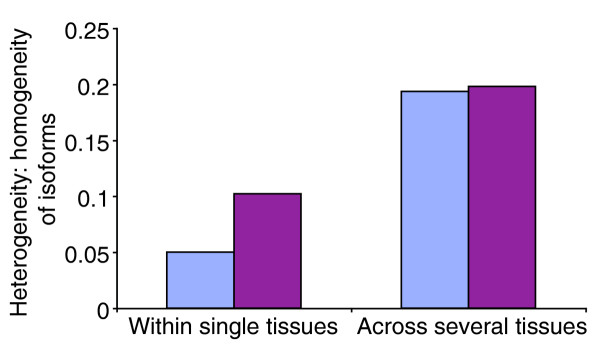
Isoforms of alternatively spliced genes are more heterogeneous across different tissues than within single tissues. The blue bars represent the ratio of all TF clusters with multiple isoforms within a tissue to all TF clusters with only one isoform within each tissue. The red bars represent the ratio of all variant clusters with multiple isoforms within a tissue to all variant clusters with only one isoform within each tissue.

We analyzed the presence of different isoforms of transcription factors within and across these 18 tissues. For this analysis we consider transcripts with coding sequence information only. We ignore variation due to 5' and 3' truncation of transcripts. We consider only cassette exons within coding sequences when assessing the differences between isoforms. Within a cluster we compute homogeneity and heterogeneity within a single tissue by checking for the transcripts from the same library and comparing the cassette exons within their coding sequences. If all transcripts from the same tissue contain the same cassette exons with same splice sites they are termed 'homogeneous within'. If the cassette exon distribution within the coding sequences of these transcripts differ, they are termed 'heterogeneous within'. We compute 'homogeneity across' and 'heterogeneity across' tissues in the same way by taking into account transcripts within the same clusters but from different libraries. As shown in Figure 5, when heterogeneity to homogeneity ratios are compared within and across tissues, there is significantly more heterogeneity of isoforms across tissues than within a single tissue (*p*-value = 0.04). This is true for both transcription factors and the rest of the genes in the mouse transcriptome.

**Figure 6:**
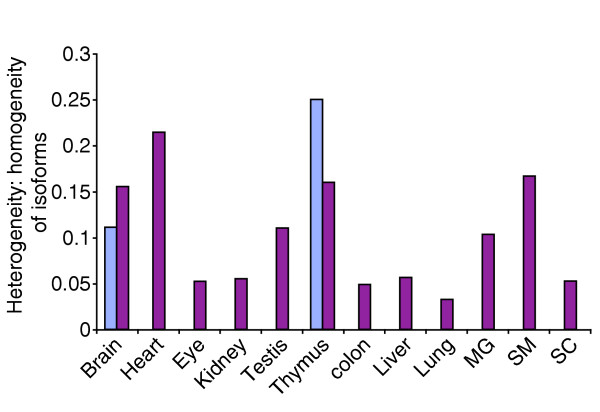
Heterogeneity versus homogeneity of isoforms in single tissues. The blue bars represent the ratio of TF clusters with multiple isoforms within the given tissue to TF clusters with only one isoform within that tissue. The red bars represent the ratio of variant clusters with multiple isoforms within the given tissue to variant clusters with only one isoform within that tissue. Tissue abbreviations: MG, mammary gland; SM, skeletal muscle; SC, spinal cord.

When single tissues are taken into account, TFs are more homogenous within each tissue analyzed. As shown in Figure 6, heterogeneity to homogeneity ratios in all tissues are lower than 1, indicating that these tissues are more homogeneous in terms of TF isoforms. In fact, except for brain and thymus, all values for TFs are zero, hence the absence of blue bars from Figure 6. When all genes are considered, heterogeneity to homogeneity ratios are also below 1, indicating homogeneity of isoforms of all genes within these tissues. However, there is still a significant difference in heterogeneity to homogeneity ratios between TF isoforms and isoforms of all genes: TFs are significantly more homogeneous within single tissues when compared to all genes (*p*-value = 0.02). (The data used in calculating the homogeneity and heterogeneity values can be found on our web page [19].)

Figures 5 and 6 show that the majority of TF isoforms and the isoforms of all alternatively spliced genes differ across tissues: within a given single tissue there generally is only one isoform. These data indicate the presence of tissue-specific alternative splicing throughout the mouse transcriptome. In addition, our findings indicate expression of different TF isoforms in different tissues. This implies contribution of alternative splicing to regulation of gene expression in a tissue-specific manner by controlling activation or repression of different sets of genes in different tissues via variant TF isoforms. These data have significant implications in further understanding the regulation of tissue-specific gene expression and control of transcription.

## Discussion

Through integrated analyses of DNA and protein sequences for TF genes, we show that alternative splicing of TFs are more prevalent in the entire mouse transcriptome and in specific tissues when compared to alternatively spliced forms of all the genes. In 78% of the tissues analyzed, higher proportions of TFs exhibit alternative splicing compared to all the genes in the mouse transcriptome. This result, along with the finding that 62% of TF loci are variant, indicates the widespread impact of alternative splicing on regulation of TF function.

We also show that alternative splicing changes TF structure by adding or deleting domains. This study reveals that 80% of alternatively spliced TFs have different domain architectures due to introduction of an in-frame cassette exon by alternative splicing. Of the altered domains, 75% have a role in DNA binding. These findings provide quantitative evidence for the role of alternative splicing in controlling the presence of domains in the proteins. They also suggest that alternative splicing might regulate TF activity by changing the architecture of the DNA-binding domains of these proteins.

Our analyses revealed that within a single tissue there generally is only one TF isoform, and that across tissues, isoforms differ. This finding indicates tissue specificity of alternatively spliced TFs and suggests that TFs might regulate gene expression in a tissue-specific manner by having different isoforms in different tissues. These findings further indicate the role of alternative splicing in regulation of tissue-specific gene expression. Activation and repression of different sets of genes within different tissues can be regulated through variant TF isoforms created by alternative splicing. These findings will significantly aid further understanding of control of transcription and tissue-specific gene expression. In addition, our study shows that all variant loci in the mouse transcriptome display isoform homogeneity within single tissues and heterogeneity across tissues. This finding demonstrates the presence of tissue-specific alternative splicing across the mouse transcriptome and greatly expands the knowledge on the tissue specificity of alternatively spliced genes.

## Conclusions

Overall, our study provides quantitative evidence for the effect of alternative splicing on protein structure and sheds light on how alternative splicing might regulate transcription factor function in a tissue-specific manner. This, in turn, reveals the contribution of alternative splicing to regulation of gene expression via tissue-specific TF isoforms. The work described here implies that future high-throughput screens of gene expression analyses should be sensitive to multiple alternatively spliced forms of TFs. Because gene-expression arrays are intended to measure transcription, the next generation of arrays should contain probes specific to all known isoforms of genes represented on the arrays. Given that alternatively spliced exons are highly conserved across species [21,22], it would be of further interest to extend this study to other organisms. Strong sequence homology between mouse, human and rat exons suggests that a comparative analysis of human, mouse and rat TF variations will be a natural extension of the studies described here.

## Materials and methods

### Development of the alternative splicing database MouSDB3

For this analysis, we constructed a database of alternatively spliced mouse transcripts called MouSDB3 [12], using the methods described in [23]. Briefly, full-length transcript nucleotide sequences were obtained by an Entrez query on 5 August 2003 from GenBank [24] with molecule selected as mRNA and limits used to exclude expressed sequence tags (ESTs), sequence-tagged sites (STSs), genome sequence survey (GSS), third-party annotation (TPA), working draft and patents. EST sequences were downloaded on 31 July 2003 from dbEST [25] by extracting only *Mus musculus *entries. All expressed sequences were mapped to a region of the University of California Santa Cruz (UCSC) February 2003 version mm3 of the mouse genome assembly using BLAT [26]. BLAT tools gfServer and gfClient were installed from jksrc444 dated 15 July 2002 [27]. This was followed by a careful alignment by SIM4 [28] version 3/3/2002 to establish splice sites of exons. A post-processing analysis computed genomic exons and determined types of variation for each exon, transcript and locus.

### Cassette exon analysis

We identified in-frame cassette exons and extracted from MouSDB3 nucleotide and amino-acid sequences for transcripts containing these exons. The selected amino-acid sequences were then analyzed with SMART [29,30] to compute protein-domain architecture for each transcript within a cluster.

### Tissue distribution of alternatively spliced TFs

From MouSDB3, we then extracted library information for the transcripts within clusters and their annotations. We used these data to compute the tissue distribution of variant transcripts as reported in Results. All scripts and README files used to carry out this data-gathering process are available upon request from the Laboratory of Computational Genomics of The Rockefeller University.

## Figures and Tables

**Table 1:** Cluster analyses of transcription factors and entire MouSDB3

	Transcription factors	Entire MouSDB3
Total number of clusters	461	55,087
Number of invariant clusters	174 (38%)	39,273 (71%)
Number of variant clusters	287 (62%)	15,814 (29%)
